# The effect of depressive symptomatology on the association of vitamin D and sleep

**DOI:** 10.1186/s12888-021-03176-4

**Published:** 2021-04-06

**Authors:** Roland Mergl, Ezgi Dogan-Sander, Anja Willenberg, Kerstin Wirkner, Jürgen Kratzsch, Steffi Riedel-Heller, Antje-Kathrin Allgaier, Ulrich Hegerl, Christian Sander

**Affiliations:** 1 Institute of Psychology, Universität der Bundeswehr München, Neubiberg, Germany; 2 Department of Psychiatry and Psychotherapy, University of Leipzig Medical Centre, Semmelweisstrasse 10, D-04103 Leipzig, Germany; 3 Institute of Laboratory Medicine, Chemistry and Molecular Diagnostics, University of Leipzig Medical Center, Leipzig, Germany; 4 LIFE - Leipzig Research Center for Civilization Diseases, University of Leipzig, Leipzig, Germany; 5 Institute for Medical Informatics, Statistics and Epidemiology (IMISE), University of Leipzig, Leipzig, Germany; 6 Institute of Social Medicine, Occupational Health and Public Health, University of Leipzig, Leipzig, Germany; 7 Department of Psychiatry, Psychosomatics, and Psychotherapy, Goethe University Frankfurt, Frankfurt am Main, Germany

**Keywords:** Vitamin D, Depression, Sleep, Actigraphy, LIFE health adult, Mediation and moderation

## Abstract

**Background:**

Sleep disorders and vitamin D deficiency are highly prevalent health problems. Few studies examined the effect of vitamin D concentrations on objectively measured sleep with high methodological quality and temporal proximity. Previous analysis within the LIFE-Adult-Study suggested that a lower concentration of serum vitamin D was associated with both shorter and later night sleep. However, no conclusion about underlying mechanisms could be drawn. We addressed the question whether this relationship is explained by the presence of depressive syndromes, which are linked to both vitamin D deficiency and sleep disturbances.

**Methods:**

It was investigated whether the association of vitamin D concentrations and night sleep parameters is mediated or moderated by depressive symptomatology. We investigated a subset (*n* = 1252) of the community sample from the LIFE-Adult-Study, in which sleep parameters had been objectively assessed using actigraphy, based on which two sleep parameters were calculated: night sleep duration and midsleep time. Serum 25(OH) D concentrations were measured using an electrochemiluminescence immunoassay. Depressive symptomatology was evaluated with the Centre for Epidemiological Studies Depression Scale. The mediation effect was analyzed by using Hayes’ PROCESS macro tool for SPSS for Windows.

**Results:**

The depressive symptomatology was neither significantly associated with night sleep duration nor midsleep time. The associations between vitamin D concentrations and night sleep duration/midsleep time through mediation by depressive symptomatology were not significant. Corresponding moderator analyses were also non-significant.

**Conclusion:**

The associations between vitamin D concentrations and night sleep parameters (sleep duration and midsleep time) seem to be neither mediated nor moderated by depressive symptomatology.

**Supplementary Information:**

The online version contains supplementary material available at 10.1186/s12888-021-03176-4.

## Background

Vitamin D is a steroid hormone playing an important role in both calcium-phosphorus and bone homeostasis [1]. Moreover, vitamin D has strong effects on brain development and adult brain function and there are close links between low levels of vitamin D and neuropsychiatric disease [2]. According to a recent review [3], vitamin D deficiency is associated with many acute and chronic diseases including periodontitis, autoimmune disorders, infectious and cardiovascular diseases, cancer, type 2 diabetes and neurological diseases. The discovery of vitamin D receptors in different cerebral areas (e.g., central gray matter in the midbrain, substantia nigra, nucleus tractus optici, nucleus interstitialis striae terminalis) suggested associations between vitamin D concentrations and mental health. High prevalence of vitamin D deficiency and insufficiency has been found in subjects with mental disorders [4–6]. Furthermore, low levels of 25-hydroxyvitamin D (25(OH) D) in patients suffering from depressive disorders were well documented (for reviews see [7, 8]) and could be replicated in several more recent studies (e.g., [9–11]). Moreover, Zhu et al. [12] found a significant inverse association of vitamin D concentrations and the severity of depressive symptoms in patients with major depressive disorder. However, there are less intervention studies examining the preventive effects of vitamin D supplementation [13]. A moderate intake of fruits/vegetables and calcium/high vitamin D sources has been shown to predict lower likelihood of depression in men [14], and subjects reporting supplementation with vitamin D had a reduced depression risk in a cross-sectional study [15]. However, in a prospective study with chronic dialysis patients with depression, calcitriol supplementation did not enhance depressive symptoms [16] and a two-sample Mendelian randomization study [17] also revealed no causal effects of serum 25(OH) D concentration on depressive symptoms or broad depression.

The influence of vitamin D deficiency on sleep has also been investigated. Sleep problems were associated with low vitamin D levels [18–20] and vitamin D supplementation has been shown to improve sleep disorders [21]. However, only few studies examined the association of vitamin D concentrations and objectively measured sleep via actigraphy so far [22–24]. Although a higher probability for short sleep (< 5 h) and low sleep efficiency (< 70%) were found to be associated with lower vitamin D concentrations [22], Bertisch and collegues could not demonstrate this association in adjusted models [23] including age, race/ethnicity, sex, and various other covariates. However, these findings were only present in older populations [22, 23]; moreover in the study of Massa and colleagues [22] the sample comprises only male participants. Using data from a large German population-based cohort study (LIFE-Adult-Study, [25]) our group could demonstrate that higher vitamin D concentrations in the general population are significantly associated with longer and earlier night sleep as assessed by actigraphic measures – even if the life-time clinical diagnosis of depressive disorders had been considered as covariate [24]. These findings were mainly in line with the previous studies examing the association of vitamin D levels and objective sleep parameters. Contrary to previous findings, we found no significant association between sleep problems assessed by PSQI (Pittsburgh Sleep Quality Index) and ESS (Epworth Sleepiness Scale).

Despite of body of evidence showing complex relationship between sleep and vitamin D the trilateral relations between serum 25(OH) D concentrations, night sleep and current depressive symptomatology remained unclear. Depressive symptomatology could be a possible mediator or moderator for the association of vitamin D and night sleep parameters. First, night sleep disturbances are part of the depressive symptomatology. Second, there are findings suggesting vitamin D deficiency in patients suffering from depression [7] and a significant negative association of vitamin D concentrations and depressive symptomatology [12]. These findings are related to patients suffering from depressive disorders; however, there is a lack of corresponding results for the total spectrum of depressive symptomatology in the general population.

The present re-analysis of data used in our previous publication [24] aimed to examine the associations between vitamin D concentrations, night-sleep parameters and depressive symptomatology in an exploratory way using a largely identical, but slightly expanded sample. It will be explored whether the association of vitamin D concentrations and night sleep parameters (night sleep duration and midsleep time) is mediated or moderated by depressive symptomatology. In view of of the existing literature it was assumed that the direction of the association goes from vitamin D concentrations over depressive symptomatology to night sleep.

## Methods

### Database and study population

The database belonged to the research project ‘LIFE’ (Leipzig Research Center for Civilisation Diseases). Within the LIFE-Adult-Study [25], recruitment of a population-based sample comprising 10.000 participants (age: 18–79 years) took place between August 2011 and November 2014 in Leipzig, Germany. One of the main aims of the LIFE-Adult-Study was to detect both the prevalence and incidence of frequent diseases (especially cardiovascular diseases, metabolic disorders, disturbances of cognition and brain functions, depressive disorders, sleep disorders, degenerative diseases of the retina, allergies and decreased immune competence) as well as subclinical disease phenotypes. In this context, standardized interviews were performed to collect sociodemographic characteristics and medical history; physical and medical examinations (like anthropometric measurements) as well as laboratory tests (for example blood sampling) took also place. Moreover, as an optional add-on assessment an actigraphy device was worn for 1 week by an arbitrary subgroup of the LIFE sample. As long as free actigraphy devices were available in the study center on the respective examination day, all LIFE-participants had been asked about their willingness to participate and had agreed or declined according to their own convenience.

For this study, those records were retrieved from the LIFE database, that belonged to LIFE-subjects that had participated in both, the blood sampling based upon which the vitamin D levels were determined as well as the actigraphic examination (*N* = 2681). Analogous to [24], the exclusion criteria were as follows: Serum-levels of 25(OH) D below limit of quantification; missing socioeconomic data; missing BMI measurement; more than 15 days between blood collection for 25(OH) D measurement and actigraphy monitoring; reported shift-work during the actigraphy period; serious medical conditions (participants with either a lifetime history of: Parkinson syndrome, intermittent claudication, autoimmune diseases, Crohn’s disease, ulcerative colitis, renal insufficiency, multiple sclerosis, requiring dialysis, liver cirrhosis, HIV; or within the last 12 month incidence of: cancer, myocardial infarction, stroke, tuberculosis, hepatitis and highly dangerous alcohol consumption (> 80 g/day for women, > 120 g/day for men)); medication with sedative-hypnotic drugs or opioids at the day of blood sampling; actigraphic data not meeting evaluation standards (see methods section).

Although the inclusion and exclusion criteria were chosen in accordance to our previous publication [24], yet a slightly larger sample could be included in the analyses presented here, due to interim re-evaluation of previously unusable data sets. Therefore, fewer data sets had to be excluded due to unsuitable actigraphy data and the final sample consisted of 1252 adults (605 males and 647 females).

### Assessment procedures

#### Serum 25(OH) D analyses

Blood withdrawal was realized according to the study protocol [25]. Serum vitamin D total concentrations were measured in accordance with the manufacturer’s protocol on an automated laboratory analyzer Cobas 8000 e602 (Roche Diagnostics, Mannheim Germany) with an electrochemiluminescence immunoassay (ECLIA) based on competition principle (Roche Diagnostics Mannheim Germany). Traceability of this method was standardized against LC-MS/MS [26] and LC-MS/MS against the NIST standard [27]. The limit of quantitation (LoQ) was 5 ng/ml. The primary measurement range was 5–70 ng/ml. Samples with concentrations > 70 ng/ml were diluted manually 1:2 using Diluent Universal (Roche Diagnostics, Mannheim, Germany).

#### Actigraphy

Within the LIFE study, actigraphic examinations were performed with SenseWear Pro 3 devices, which allow recording of multiple sensor parameters (2-axis body acceleration, temperature of the skin, heat flux and galvanic skin response). The SenseWear was successfully validated against polysomnography and is characterized by accurate detection of several sleep parameters (total sleep time, sleep efficiency, wake after sleep onset [28–32], sleep onset and sleep offset [33, 34]).

Participants were instructed to wear SenseWear Pro 3 devices for seven consecutive days and to keep a diary regarding own activities and bedtime periods (by making a note of ‘lights off time’ and ‘get up time’). Basic evaluations of the recorded data were performed using the SenseWear Professional 6.1 software package (BodyMedia; Pittsburgh, Pennsylvania), which contains specific algorithms to classify periods of 1 min as either being up or lying down (‚rest‘ periods`) and wake or asleep (‚sleep‘ periods`). In addition, the software detects temporary removal (off-arm periods) of the device automatically.

Following automatic sleep and rest classification, exporting of data to a Microsoft Excel template with Visual Basic for Applications (VBA) macros was performed, in order to customize analysis windows to the day-night-cycles of the study participants instead of using the fixed time windows sleep analyses used within the SenseWear software (for further details see [24]). In short, based on the bedtime information given in the sleep diaries the corresponding night sleep intervals (NSI) were identified manually for each subject. Correspondingly, a daytime interval (DTI) was defined as the period between two consecutive NSIs. Therefore, “days” are defined as intervals from the midpoint of a NSI to the midpoint of the subsequent NSI, whereas a night-day-cycle (NDC) is defined by adding a NSI with the subsequent DTI. Standard actigraphy parameters were calculated for all NSI, DTI and NDC. For this study, the following sleep parameters were used, as an association with vitamin D could be shown in our previous publication [24]:

- Night sleep duration (sum of all minutes classified as ‘sleep’ within a NSI);
- Midsleep time (half time between sleep onset and offset as detected by using the SenseWear algorithm within a NSI).

For further analyses, actigraphic results were individually averaged across the corresponding measuring days. For this, only NSIs without off-arm periods, and DTIs of ‘days’ with a minimum duration of 20 h and an off-wrist duration of less than 15% of the DTI were considered. Data sets were included in analyses when they did contain a) at least four NSI during the working week and at least one weekend NSI, and b) at least five complete NDCs.

### Questionnaires & interviews

During the baseline visit at the LIFE-Adult-study center in Leipzig data on demographic and socioeconomic factors, medical history, current medication as well as well-being were gathered by using questionnaires and standardized interviews; for this purpose, trained raters were employed [25].

The presence of current depressive symptoms (within the last week) was assessed by a self-report questionnaire, the German version of the Centre for Epidemiological Studies Depression Scale (CES-D, [35, 36]). This scale represents a reliable and valid tool for detecting depressive symptomatology and has been applied in the general population [37, 38]. The CES-D comprises 20 items and the sum score has a range from 0 to 60, with higher total scores reflecting more depressive symptoms and a sum score ≥ 23 being used as indicator of clinically relevant depressive symptoms [38–40]. We computed a modified CES-D sum score by removing the item 11 (representing sleep problems) from the original CES-D total score. This procedure was relevant in view of the intended correlation of a CES-D score with night sleep parameters. Otherwise, it would not have been possible to reduce collinearity.

Based on data regarding education, the occupational status and the equivalent household income a multidimensional index of socioeconomic status was computed [41, 42]. Body weight was measured by means of an electronic scale and body height by means of a stadiometer in order to compute the body mass index (BMI). Information on medical history was collected by questions regarding the occurrence of selected diseases (lifetime occurrence, occurrence within the last year and current treatment). Medication taken in the last week before the baseline visit (including vitamin D supplements) was documented by using the ATC (Anatomical Therapeutic Chemical) classification system. Alcohol consumption within the last 12 months was assessed by making a note of the quantity and frequency of alcoholic beverages [25].

### Statistical analyses

Demographical characteristics, clinical variables and actigraphic parameters were analyzed by using descriptive statistics dependent from the corresponding scale level. For the assessment of the trilateral relations between vitamin D concentrations, current depressive symptomatology (according to the CES-D sum score) and night sleep parameters (night sleep duration and midsleep time) Spearman-Brown correlation coefficients were chosen.

To test the mediation models for night sleep duration and midsleep time, Hayes’ PROCESS macro tool for SPSS for Windows, based on the mediation method with 10,000 bootstrap bias-corrected 95% confidence intervals, was chosen as recommended by Hayes [43] as measures of indirect effects. If they do not include zero, the indirect effect can be regarded as being statistically significant [44]. We selected two mediation models: The first model did not include any covariates; in the second model, three variables (age, the BMI score and the season in which vitamin D concentrations had been measured) were treated as covariates. In the mediation models, the vitamin D concentration was the independent variable, the CES-D sum score was the mediator and night sleep parameters (night sleep duration and midsleep time, respectively) represented the dependent variables.

For moderator analyses, linear regression analyses were chosen. The dependent variables were night sleep duration and midsleep time, respectively. The independent variables in these models were vitamin D concentration, the CES-D sum score (without item 11) and the corresponding interaction term. We chose two models: The first model did not include any covariates; in the second model, three variables (age, the BMI score and the season in which vitamin D concentrations had been measured) were selected as covariates.

All statistical analyses were done by using the SPSS software version 25.0 for Windows. The significance level was set at α = 0.05 (two-tailed).

## Results

The characteristics of the final sample are shown in Table 1. The mean serum 25(OH) D concentration in the sample (23.44 ng/ml; SD: 11.15 ng/ml) reflects vitamin D insufficiency [45]. The mean night sleep duration was rather low (06:16 hh:mm; SD: 00:59 hh:mm). According to the original CES-D sum scores, depressive symptomatology was present in 16.9% of the final sample.

**Table 1:** Characteristics of the final sample

Variable	Total sample	Men	Women
*N (%)*	1252 (100%)	605 (48.3%)	647 (51.7%)
*Age (years), means (SD)*	57.09 (12.28)	58.39 (12.19)	55.89 (12.25)
*Birth, n (%)*			
In Germany	1196 (95.5%)	574 (94.9%)	622 (96.1%)
In another country	56 (4.5%)	31 (5.1%)	25 (3.9%)
*Family status, n (%)*			
Married, living together	787 (62.9%)	419 (69.3%)	368 (56.9%)
Married, not living together	28 (2.2%)	9 (1.5%)	19 (2.9%)
Single	209 (16.7%)	93 (15.4%)	116 (17.9%)
Divorced	163 (13.0%)	68 (11.2%)	95 (14.7%)
Widowed	65 (5.2%)	16 (2.6%)	49 (7.6%)
*Socio-economic status, n (%)*^a^			
High	339 (27.1%)	180 (29.8%)	159 (24.6%)
Middle	740 (59.1%)	341 (56.4%)	399 (61.7%)
Low	171 (13.7%)	83 (13.7%)	88 (13.6%)
*Employment status, n (%)*			
Gainful employment	708 (56.6%)	328 (54.2%)	380 (58.7%)
Unemployment	99 (7.9%)	48 (7.9%)	51 (7.9%)
Retirement	445 (35.5%)	229 (37.9%)	216 (33.4%)
*BMI, means (SD)*	27.20 (4.49)	27.70 (3.89)	26.73 (4.94)
*Consumption of alcohol (g/day)*,			
*means (SD)*^*b*^	12.88 (17.81)	19.47 (21.34)	6.70 (10.44)
*Vitamin D supplement, n (%)*	225 (18.0%)	76 (12.6%)	149 (23.0%)
*Season in which vitamin D concen-trations had been measured, n(%)*			
Winter	298 (23.8%)	160 (26.4%)	138 (21.3%)
Spring	304 (24.3%)	146 (24.1%)	158 (24.4%)
Summer	329 (26.3%)	157 (26.0%)	172 (26.6%)
Autumn	321 (25.6%)	142 (23.5%)	179 (27.7%)
*Lifetime diagnosis of depressive disorders, n (%)*	132 (10.5%)	41 (6.8%)	91 (14.1%)
*Lifetime diagnosis of diseases of the thyroid gland, n(%)*	322 (25.7%)	83 (13.7%)	239 (36.9%)
*Depression status according to the CES-D sum score, n (%)*			
Clinically inconspicuous symptomatology (score: 0–14)	1041 (83.1%)	525 (86.8%)	516 (79.8%)
Mild to moderate depressive symptomatology, clinically relevant (score: 15–21)	139 (11.1%)	55 (9.1%)	84 (13.0%)
Severe depressive symptomatology, possibly associated with Major Depression (score: ≥22)	72 (5.8%)	25 (4.1%)	47 (7.3%)
*Serum 25(OH) D concentration (ng/ml), means (SD)*	23.44 (11.15)	23.56 (11.00)	23.32 (11.29)
*Night sleep duration (hh:mm), means (SD)*	6:16 (0:59)	6:03 (0:59)	6:28 (0:56)
*Mid sleep time (hh:mm), means (SD)*	03:10 (0:50)	03:14 (0:51)	03:07 (0:49)

Notes: CES-D: German version of the Centre for Epidemiological Studies Depression Scale (CES-D, [35, 36]); *ml* milliliter, *N/n* sample size, *ng* nanogram, *SD* standard deviation

^a^Reduced N due to 2 missings (total sample: 1250, males: 604, females: 646),

^b^Reduced N due to 1 missing (total sample: 1251, males: 605, females: 646)

Table 2 summarized the trilateral associations between serum 25 (OH) D concentrations, CES-D sum scores (after removing item 11) and night sleep parameters (night sleep duration, midsleep time). Only one association was found to be statistically significant: the positive correlation of 25(OH) D concentrations with night sleep duration (rho = 0.08; *p* = 0.006).

**Table 2:** Correlation coefficient matrix of serum 25(OH) D concentrations, CES-D sum scores (after removing item 11 (sleep problems)) and night sleep parameters (night sleep duration, midsleep time) in the final sample (*N* = 1252)

	CES-D sum score	Serum 25 (OH) D con-centration	Night sleep duration	Midsleep time
*CES-D sum score*	1	−0.05	0.02	−0.01
*Serum 25(OH) D concentration*	–	1	0.08**	−0.02
*Night sleep duration*	–	–	1	− 0.02
*Midsleep time*	–	–	–	1

Notes: CES-D: German version of the Centre for Epidemiological Studies Depression Scale (CES-D, [35, 36]) without item 11 representing intensity of sleep problems; Spearman-Brown correlation coefficients are given

^**^p = 0.01

**Fig. 1:**
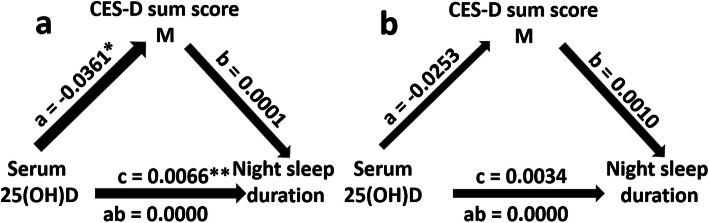
Mediation model (**1a**: without covariates; **1b**: with covariates (age, the BMI score and the season in which vitamin D concentrations had been measured)) showing both the direct effect of serum 25(OH) D concentrations on night sleep duration (path coefficient c) and the indirect effect of serum 25(OH) D concentrations on night sleep duration mediated through depressive symptomatology (as measured by modified CES-D sum scores) (path coefficient ab) in *N* = 1252 individuals of the study sample. The figure depicts the unstandardized path coefficients (a,b,c and ab). CES-D: German version of the Centre for Epidemiological Studies Depression Scale (CES-D, [35, 36]) without item 11 representing intensity of sleep problems; serum 25(OH)D: serum 25(OH) D concentration. * *p* < 0.05

As shown in Fig. 1a, the direct effect of serum 25(OH) D concentrations on night sleep duration in the simple mediation model without covariates (c = 0.007; *p* = 0.009) was significant. The same was true for the effect of serum 25(OH) D concentrations on CES-D sum scores in this model (a = − 0.036; *p* = 0.021) whereas the effect of the CES-D sum scores on night sleep duration failed to be significant (b = − 0.0001; *p* = 0.978). The indirect effect of serum 25(OH) D concentrations on night sleep duration via depressive symptomatology was non-significant, too (ab = 0.000; 95% CI [− 0.0004, 0.0003]). In the corresponding mediation model with covariates (age, the BMI score and the season in which vitamin D concentrations had been measured) all afore-mentioned effects failed to be statistically significant (see Fig. 1b).

**Fig. 2:**
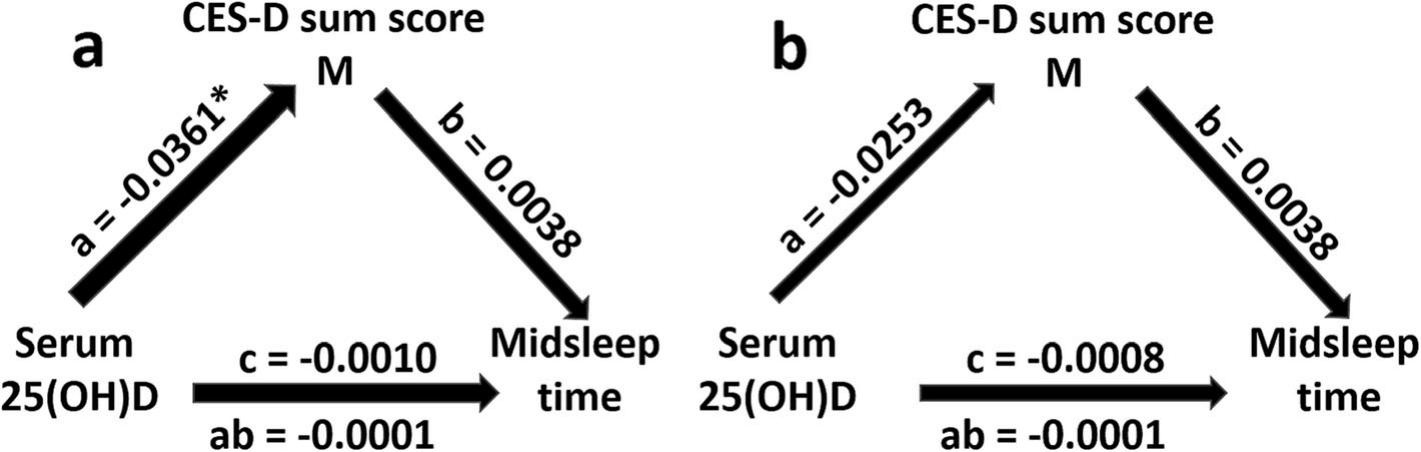
Mediation model (**2a**: without covariates; **2b**: with covariates (age, the BMI score and the season in which vitamin D concentrations had been measured)) showing both the direct effect of serum 25(OH) D concentrations on midsleep time (path coefficient c) and the indirect effect of serum 25(OH) D concentrations on midsleep time mediated through depressive symptomatology (as measured by modified CES-D sum scores) (path coefficient ab) in *N* = 1252 individuals of the study sample. The figure depicts the unstandardized path coefficients (a,b,c and ab). CES-D: German version of the Centre for Epidemiological Studies Depression Scale (CES-D, [35, 36]) without item 11 representing intensity of sleep problems; serum 25(OH)D: serum 25(OH) D concentration. * *p* < 0.05

Figure 2a demonstrates that the direct effect of serum 25(OH) D concentrations on midsleep time in the simple mediation model without covariates was negative, but non-significant (c = − 0.001; *p* = 0.641). Instead, there was a significant negative effect of serum 25(OH) D concentrations on CES-D sum scores in this model (a = − 0.0363; *p* = 0.021) whereas the effect of the CES-D sum scores on midsleep time failed to be significant (b = 0.004; *p* = 0.325). The indirect effect of serum 25(OH) D concentrations on midsleep time via CES-D sum scores was non-significant, too (ab = − 0.0001; 95% CI [− 0.0005, 0.0002]). In the corresponding mediation model with covariates (age, the BMI score and the season in which vitamin D concentrations had been measured) all above-mentioned effects were non-significant (see Fig. 2b).

According to moderator analyses, the effects of the interaction of the factors “vitamin D concentration” and “CES-D sum scores without item 11 (sleep problems)” on night sleep duration failed to be significant. The same was true for corresponding regression models with midsleep time as dependent variable (see Table S1 in Supplement S2).

## Discussion

The main aim of this study was to examine the trilateral associations between serum vitamin D concentrations, current depressive symptomatology and night sleep duration in the general population of a German city as a follow-up study to our previous findings [24]. In addition, it was investigated whether vitamin D concentrations had significant effects on night sleep duration and midsleep time (as suggested by [24]) through the mediation of depressive symptomatology and whether it represents a relevant moderator variable for this association. According to the main results of this study, serum 25(OH) D concentrations did not have an indirect effect on night sleep duration and midsleep time through a mediation effect of depressive symptomatology: There was a significantly negative association of vitamin D concentrations with depressive symptomatology in the simple mediation models as expected due to previous findings suggesting a close link between depression and low levels of serum 25(OH) D [7–12]. However, the sum score in the CES-D (after removing the item concerning sleep problems) was neither significantly associated with night sleep duration nor midsleep time which would have been required for a mediator between vitamin D concentrations and night sleep.

In the simple mediation model (without any covariates), serum 25(OH) D concentrations had a direct and significantly positive effect on night sleep duration in a German community sample. This finding was in line with previous studies, in which sleep parameters had been measured in an objective way by using actigraphy and a positive association between serum 25(OH) D concentrations and night sleep duration had been found [22–24]). However, this association was found to be not mediated by depressive symptomatology and failed to be significant if the effects of relevant covariates (age, the BMI score and the season in which the vitamin D concentrations had been measured) were controlled for. Thus, a specific effect of vitamin D concentrations on night sleep duration seems to be quite improbable.

Another picture is present for the trilateral associations between serum 25(OH) D concentrations, current depressive symptomatology and midsleep time: Midsleep time was neither significantly associated with serum 25(OH) D concentrations nor with the sum score in the CES-D scale. In line with these findings, vitamin D concentrations had neither significant direct nor indirect effects on midsleep time in our sample. This result was unexpected since in another study using a very similar sample [24] an inverse significant association between midsleep time and serum 25(OH) D concentrations had been reported. In our sample, this association was also negative, but failed to be significant. The discrepancies might be due to different statistical approaches (multiple linear regression analyses with centered vitamin D concentrations [24] versus mediation and correlation analyses in the present study). Differences between this study and our previous publication [24] regarding mean vitamin D concentrations (23.4 ng/ml versus 21.9 ng/ml), mean night sleep duration (376 min versus 379 min), mean midsleep time (03:10 (hh:mm) versus 03:08 (hh:mm)) and the proportion of patients with a lifetime history of depressive disorders (10.5% versus 9.7%) cannot sufficiently explain these differences.

Further analyses revealed that the association between vitamin D concentrations on the one hand and night sleep parameters (night sleep duration and midsleep time) on the other hand was not moderated by the severity of depressive symptoms (without sleep problems).

Overall, the literature about sleep problems, depression and vitamin D is scarce with large methodological heterogeneity. Sleep problems are in most cases measured only subjectively via questionnaires, thus limiting the comparability wth our results, since objective sleep parameters were used for this mediation analysis. In a case series with depressed adolescents, an improvement in well-being and other depressive symptoms, including sleep difficulties, was shown after vitamin D supplementation [46]. In a recent meta-analysis [47], it has been demonstrated that vitamin D supplementation decreased depressive symptoms as well as sleep problems among patients with psychiatric diseases. Furthermore, Fallah and colleagues showed in a systematic review, that sleep quality and postpartum depression are associated to vitamin D deficiency [48]. In our study using a community sample we failed to show a significant association between night sleep and midsleep time and vitamin D in the mediation models with covariates. Furthermore, no association between depressive symptoms and night sleep and midsleep time was found. This is in line with a study where no effect of daily vitamin D supplementation for a year on depressive symptoms and sleep quality was found among overweight and obese postmenopausal women [49].

In this study, we hypothesized that depression might have a mediator effect on the relationship of night sleep and vitamin D levels according to the previous findings reporting vitamin D deficiency as a risk factor for depression and sleep disorders [50–52]. However, a reverse association is also conceivable. It could be assumed that a sunlight-avoiding lifestyle as well as lack of physical activity caused by a depressive disorder can play a role in both vitamin D deficiency and sleep disorders. However, since vitamin D deficiency is also associated with low physical performance [45] and fatigue in several patient groups (e.g. [53]), an only monocausal association cannot be drawn based on existing literature.

The present study has some methodological limitations: First, the data were collected within a cross-sectional cohort study; thus, the temporal associations between vitamin D concentrations, depressive symptomatology and night sleep parameters could not be examined in an adequate manner. Second, although the mediation analyses considered relevant covariates (age, the BMI score and the season in which the vitamin D concentrations had been measured) we cannot exclude the possibility that other potential covariates had a significant effect on the night sleep outcomes (night sleep duration and midsleep time), too. Third, only 12.5% of the total sample of the Life-Adult-Study could be included in the present study. The reason for this was that actigraphy was only an optional additional examination, so that in total both vitamin D level and actigraphy data were only available for about ¼ of the 10,000 LIFE participants. Although the examination was offered in principle, selection effects cannot be excluded, e.g., subjects who had experienced sleep disturbances might have been more approachable, as might subjects who were more physically active and interested in quantifying their physical activity. To make matters worse, after all exclusion criteria were taken into account, only just under half of the available data sets could actually be included in the analysis. Again, bias cannot be ruled out; however, the selection criteria were defined with the intention of excluding disease- or medication-related changes in sleep. Fourth, it must be mentioned that lack of statistical significance regarding an association is not a proof of absence of that association because the negative finding could have been due to a type II statistical error. Fifth, the CES-D sum score might have shortcomings as a measure of depression. It would have been interesting to use a more comprehensive inventory of depressive symptoms. Major strengths of this study include a large sample size with the study population being not restricted to a certain age group or gender. Thus, the external validity of the findings was quite high. The interval between the measurement of vitamin D levels and the assessment of night sleep parameters was rather short (maximally 15 days). For the measurement of vitamin D concentrations and sleep parameters, high-quality methods had been used.

## Conclusion

In conclusion, the associations between vitamin D concentrations and night sleep duration as well as midsleep time seem to be neither mediated nor moderated by depressive symptomatology. It will be important to confirm this finding in the context of a large representative longitudinal cohort study. Such a study would be of special interest because the temporal associations between serum vitamin D concentrations, the intensity of depressive symptoms and night sleep parameters could be investigated within this study in an adequate way.

## Supplementary Information

**Additional file 1.** Used Items from LIFE Adult assessment program.

**Additional file 2.** Results of regression analyses.

## Data Availability

The data that support the findings of this study are available from the Leipzig Research Center for Civilisation Diseases (LIFE) but restrictions apply to the availability of these data, which were used under license (project number: Sander-2019-479-09) for the current study, and so are not publicly available. Data are however available from the authors upon reasonable request and with permission of LIFE.
