# Supplementary material for: The effect of depressive symptomatology on the association of vitamin D and sleep
Source: BMC Psychiatry. 2021 Apr 6;21:178. doi: 10.1186/s12888-021-03176-4 (PMC8025511; doi:10.1186/s12888-021-03176-4)
Supplement: Supplementary file 1 — Additional file 1. Used Items from LIFE Adult assessment program. [file 12888_2021_3176_MOESM1_ESM.pdf]

The effect of depressive symptomatology on the association of vitamin D and sleep

Supplement S1: Used Items from LIFE Adult assessment program

Roland Mergl [1]*, Ezgi Dogan-Sander [2]*, Anja Willenberg [3], Kerstin Wirkner [4,5], Jürgen Kratzsch [3,4], Steffi Riedel-Heller [4,6], Antje-Kathrin Allgaier [1], Ulrich Hegerl [7], Christian Sander [2,4]

* Both authors equally contributed to the manuscript.

[1] Institute of Psychology, Universität der Bundeswehr München, Neubiberg, Germany

[2] Department of Psychiatry and Psychotherapy, University of Leipzig Medical Center, Leipzig, Germany

[3] Institute of Laboratory Medicine, Clinical Chemistry and Molecular Diagnostics, University of Leipzig Medical Center, Leipzig, Germany

[4] LIFE - Leipzig Research Center for Civilization Diseases, University of Leipzig, Leipzig, Germany

[5] Institute for Medical Informatics, Statistics and Epidemiology (IMISE), University of Leipzig, Leipzig, Germany

[6] Institute of Social Medicine, Occupational Health and Public Health, University of Leipzig, Leipzig, Germany

[7] Department of Psychiatry, Psychosomatics, and Psychotherapy, Goethe University Frankfurt, Frankfurt am Main, Germany

The data reported in the article are taken from the LIFE Adult study, whose participants received a wide range of physical examinations, were interviewed on various topics and filled out various questionnaires as part of a very extensive research program (for details see Löffler et al. 2016[1]). Apart from the *Center of Epidemiological Studies Depression Scale* (CES-D, Radloff, 1997[2]; German Version; Hautziger et al., 2012[3]), the following items from the comprehensive LIFE assessment were used:

<u>Sociodemography (Interview):</u>

- In which country were you born?
  [Options: Germany / Other country]
- What is your marital status?
  [Options: Married, living together with spouse / Married, living separately from spouse / Single / Divorced / Widowed]
- Are you currently employed? (Employment means any paid or income-related activity, regardless of what time frame it has).
  [Options: Yes, I am a full-time employee with 35 hours or more per week / Yes, I am part-time with 15 to 34 hours per week / Yes, I am part-time/hourly employed with less than 15 hours per week / no]
- If no: Please tell me which group you belong to?
  [Options: in training or study / unemployed (recipient of unemployment benefit I) / unemployed (recipient of unemployment benefit II (Hartz IV) / unemployed (without

---

[1] Loeffler M, Engel C, Ahnert P, Alfermann D, Arelin K, Baber R, et al. The LIFE-Adult-Study: objectives and design of a population-based cohort study with 10,000 deeply phenotyped adults in Germany. BMC Public Health. 2015;15: 691

[2] Radloff LS. The CES-D Scale: A self-report depression scale for research in the general population. Appl Psychol Measurement. 1977;1: 385-401.

[3] Hautzinger M, Bailer M, Hofmeister D, Keller F. Allgemeine Depressionsskala (ADS); Manual. Hogrefe, 2012.

receiving benefits) / Housewife or -husband / Military or community service performer or voluntary social year / in maternity or parental leave / in early retirement, pension, retirement / None of the above]

- What is your highest general education level?

  [Options: still a student without a degree / Leaving school without a secondary school leaving certificate (without elementary school leaving certificate) / Secondary school certificate (elementary school certificate) / Secondary school leaving certificate ("Mittlere Reife") / Completion of the polytechnic secondary school 10th grade (before 1965: 8th grade) / Advanced technical college entrance qualification, completion of technical college / General or subject-related higher education entrance qualification ("Abitur") (Gymnasium/EOS, also EOS with apprenticeship) / another school leaving certificate]

- What is your vocational training qualification? (Multiple selection possible)

  [Options: Still in vocational training (trainee, student) / No professional qualification and I am not in vocational training / Occupational Professional training (apprenticeship) completed / Vocational school education (vocational school, commercial school) completed / Training at a technical college, completed master school, technical school, vocational or technical academy / University of Applied Sciences degree / University degree / other completed professional training]

- What is the total monthly net income of your household? (Monthly net income refers to the sum of wages, salary, income from self-employment, or pension. Please calculate also the income from public subsidies, income from renting, rent, housing allowance, child benefit and other income and then deduct taxes and social security contributions. If someone in your household is self-employed, please consider the following for this person the average net remuneration less operating expenses.)

[Options: under 150 Euro / 150 to under 400 Euro / 400 to under 500 Euro / 500 to under 750 Euro / 750 to under 1 000 Euro / 1000 to under 1250 Euro / 1250 to under 1500 Euro / 1500 to under 1750 Euro / 1750 to below 2000 Euro / 2000 to under 2250 Euro / 2250 to under 2500 Euro / 2500 to under 2750 Euro / 2750 to under 3000 Euro / 3000 to under 3250 Euro / 3250 to under 3500 Euro / 3500 to under 3750 Euro / 3750 to under 4000 Euro / 4000 to under 4500 Euro / 4500 to under 5000 Euro / 5 000 to under 5500 Euro / 5500 to under 6000 Euro / 6000 to below 7500 Euro / 7500 to under 10000 Euro / 10000 to under 20000 Euro / 20000 Euro and more]

<u>Alcohol Consumption (Food Frequency Questionnaire):</u>

- Please remember the last 12 months. How often do you drink...
  - Light beer and low-alcohol beer
  - Beer with normal or higher alcohol content
  - Wine, sparkling wine, fruit wine
  - High-proof alcoholic beverages (rum, brandy, liqueur, clear schnapps, etc.)

  [Options: Several times a day / Daily or almost daily / Several times a week / About once a week / Two to three times a month / Once a month or less / (Almost) never]
- If you take the following drinks, how much do you usually drink on these days?
  - Light beer and low-alcohol beer
  - Beer with normal or higher alcohol content
  - Wine, sparkling wine, fruit wine
  - High-proof alcoholic beverages (rum, brandy, liqueur, clear schnapps, etc.)

  [Options: More than 0,7 l / Between 0.4 and 0.7 l / Between 0.2 and 0.4 l / Between 0.1 and 0.2 l / Less than 0.1 l / Drink (almost) never]

Medical anamnesis (interview):

- Have you ever been diagnosed by a doctor with...
    - Parkinson's Syndrome
    - Claudicatio intermittens / arterial occlusive disease
    - Heart attack
    - Stroke
    - Autoimmune diseases (e.g. lupus erythematosus, Sjögren syndrome)
    - Colitis ulcerosa / Morbus Crohn
    - Renal insufficiency
    - Dialysis obligation
    - Multiple sclerosis
    - Liver cirrhosis
    - Tuberculosis
    - Hepatitis (A, B, C, D, E)
    - HIV
    - Thyroid disorders
    - Depression
    - Cancer

    [Options: yes / no / don't know]
- In the last 12 months, have you had...
    - Parkinson's Syndrome
    - Claudicatio intermittens / arterial occlusive disease
    - Heart attack
    - Stroke

- Autoimmune diseases (e.g. lupus erythematosus, Sjögren syndrome)
- Colitis ulcerosa / Morbus Crohn
- Renal insufficiency
- Dialysis obligation
- Multiple sclerosis
- Liver cirrhosis
- Tuberculosis
- Hepatitis (A, B, C, D, E)
- HIV
- Thyroid disorders
- Depression
- Cancer

[Options: yes / no / don't know]

Actigraphy (Interview):

- Did you work in a shift system while wearing the actometer?

  [Options: Yes / No]
