# Supplementary material for: The effect of depressive symptomatology on the association of vitamin D and sleep
Source: BMC Psychiatry. 2021 Apr 6;21:178. doi: 10.1186/s12888-021-03176-4 (PMC8025511; doi:10.1186/s12888-021-03176-4)
Supplement: Supplementary file 2 — Additional file 2. Results of regression analyses. [file 12888_2021_3176_MOESM2_ESM.pdf]

The effect of depressive symptomatology on the association of vitamin D and sleep

Supplement S2: Results of regression analyses

Roland Mergl [1]*, Ezgi Dogan-Sander [2]*, Anja Willenberg [3], Kerstin Wirkner [4,5], Jürgen Kratzsch [3,4], Steffi Riedel-Heller [4,6], Antje-Kathrin Allgaier [1], Ulrich Hegerl [7], Christian Sander [2,4]

* Both authors equally contributed to the manuscript.

[1] Institute of Psychology, Universität der Bundeswehr München, Neubiberg, Germany

[2] Department of Psychiatry and Psychotherapy, University of Leipzig Medical Center, Leipzig, Germany

[3] Institute of Laboratory Medicine, Clinical Chemistry and Molecular Diagnostics, University of Leipzig Medical Center, Leipzig, Germany

[4] LIFE - Leipzig Research Center for Civilization Diseases, University of Leipzig, Leipzig, Germany

[5] Institute for Medical Informatics, Statistics and Epidemiology (IMISE), University of Leipzig, Leipzig, Germany

[6] Institute of Social Medicine, Occupational Health and Public Health, University of Leipzig, Leipzig, Germany

[7] Department of Psychiatry, Psychosomatics, and Psychotherapy, Goethe University Frankfurt, Frankfurt am Main, Germany

Table S1. Results of regression analyses regarding the role of the severity of depressive symptoms as possible moderator for the association of vitamin D concentrations and night sleep parameters

Independent variables	*b [95% CI]*	*beta*	*p*
Night sleep duration as dependent variable			
1. Model without any covariates			
CES-D sum score (without item 11) (CES-D)	0.001 [-0.019;0.022]	0.009	0.892
Vitamin D concentration (Vit D)	0.007 [-0.002;0.016]	0.080	0.106
CES-D x Vit D	-0.00006 [-0.001;0.001]	-0.010	0.891
2. Model with covariates			
CES-D sum score (without item 11) (CES-D)	0.002 [-0.019;0.022]	0.010	0.872
Vitamin D concentration (Vit D)	0.004 [-0.005;0.012]	0.041	0.407
CES-D x Vit D	-0.00003 [-0.001;0.001]	-0.006	0.938
Midsleep time as dependent variable			
1. Model without any covariates			
CES-D sum score (without item 11) (CES-D)	0.007 [-0.011;0.024]	0.048	0.465
Vitamin D concentration (Vit D)	0.00004 [-0.007;0.007]	0.001	0.991
CES-D x Vit D	-0.0001 [-0.001;0.001]	-0.025	0.735
2. Model with covariates			
CES-D sum score (without item 11) (CES-D)	0.007 [-0.010;0.025]	0.052	0.425
Vitamin D concentration (Vit D)	0.00042 [-0.007;0.008]	0.006	0.913
CES-D x Vit D	-0.00015 [-0.001;0.001]	-0.031	0.681

Notes: b: unstandardized regression coefficient; beta: standardized regression coefficient; CES-D: German version of the Centre for Epidemiological Studies Depression Scale (CES-D, [35, 36]) – the corresponding sum score has been modified by subtracting item 11 (reflecting the intensity of sleep problems) form the original total score; CI: confidence interval; p: p value. The results of different linear regression models are given: models without any covariates and models with covariates (age, the BMI score and the season in which vitamin D concentrations had been measured).
